# Megalin Orchestrates FcRn Endocytosis and Trafficking

**DOI:** 10.3390/cells12010053

**Published:** 2022-12-22

**Authors:** Eileen Dahlke, Yaman Anan, Lea Maximiliane Klie, Ariane Elisabeth Hartkopf, Franziska Theilig

**Affiliations:** Institute of Anatomy, Christian Albrechts-University Kiel, 24118 Kiel, Germany

**Keywords:** megalin, neonatal Fc receptor (FcRn), endocytosis, trafficking, protein interaction

## Abstract

The neonatal Fc receptor (FcRn) is highly expressed in the renal proximal tubule and is important for the reclamation of albumin by cellular transcytosis to prevent its loss in the urine. The initial event of this transcellular transport mechanism is the endocytosis of albumin by the apical scavenger receptors megalin and cubilin. An interaction of megalin and FcRn was postulated, however, evidence is still missing. Similarly, the intracellular trafficking of FcRn remains unknown and shall be identified in our study. Using a Venus-based bimolecular fluorescence complementation system, we detected an interaction between megalin and FcRn in the endosomal compartment, which significantly increased with the induction of endocytosis using albumin or lactoglobulin as a ligand. The interaction between megalin and FcRn occurred at a neutral and acidic pH between the extracellular domains of both proteins. Amnionless, another transmembrane acceptor of cubilin, revealed no interaction with FcRn. With the induction of endocytosis by albumin or lactoglobulin, super resolution microscopy demonstrated a redistribution of megalin and FcRn into clathrin vesicles and early endosomes. This trafficking into clathrin vesicles was impaired in megalin-deficient cells upon albumin-induced endocytosis, supporting the role of megalin in FcRn redistribution. Our results indicate that megalin and FcRn specifically bind and interact within their extracellular domains. The availability of megalin is necessary for the redistribution of FcRn. Megalin, therefore, orchestrates FcRn endocytosis and intracellular trafficking as an early event intranscytosis.

## 1. Introduction

The neonatal Fc receptor (FcRn) was initially identified as responsible receptor for IgG transport across the placenta, and later for IgG recycling [1]. In the meantime, additional roles of FcRn have been discovered, including albumin recycling, the bidirectional transport of IgG and albumin across a range of polarized cellular barriers, and potentiating efficient responses to IgG-immune complexes [1]. Albumin is the most abundant serum protein, with many important physiologic functions; these include its role in the determination of colloid osmotic pressure, as an antioxidant, its pH buffering capacity, and role as a transport vehicle of endogenous and exogenous compounds. Albumin exclusively produced in the liver is reclaimed by the kidney in FcRn-mediated transcytosis. Transcytosis is a transport process for large molecules across epithelial barriers consisting of endocytosis, intracellular distribution by endosome traffic, and exocytosis at the apical or basolateral membrane, according to the direction of transport. FcRn is so far the only known transcytosis receptor for albumin [2]. In the kidney, FcRn is expressed in podocytes and in the brush border membrane and intracellular vesicles of the proximal tubule [3]. The importance of the renal proximal tubular FcRn for albumin turnover has been investigated by several groups [3,4,5]; they have shown that the kidney importantly contributes to this turnover via the reabsorption of albumin passing the glomerular filter, and thereby prevents the reduction in albumin half-life as observed in FcRn knockout mice. Significant amounts of albumin pass the glomerular filter, estimated to be approximately 1 g per day in healthy humans; less than 0.05 g per day of albumin can be detected in the final urine, although the amount of filtered and reabsorbed albumin may still be controversially discussed [6,7,8]. Endocytic activity at the brush border is a highly active process, with clathrin-mediated endocytosis as the most abundant endocytic pathway in the convoluted part of the renal proximal tubule [9]. The multiligand scavenger receptors, megalin (low-density lipoprotein-related protein 2, LRP2) and cubilin (CUBN), are expressed in the brush border membrane of the proximal tubule and are known to bind to more than 50 different plasma proteins, including albumin [10]. After ligand binding to megalin or cubilin, clathrin-mediated endocytosis will be initiated [11]. The cytoplasmic domain of megalin possesses binding motifs for disabled-2 (Dab2) for protein binding to clathrin, to ensure micropinocytosis into the apical endocytic compartment [10]. Cubilin, lacking a cytoplasmic domain, uses either megalin or amnionless (AMN)for its internalization. Likewise, AMN contains a Dab2-binding motif for micropinocytosis. Albumin, capable of binding to both scavenger receptors, dissociates from them at a pH below 6.5 [12], presumably in early endosomes during acidification. The receptors recycle back to the apical plasma membrane [13], and albumin is presented to transcytose back into the blood stream in an intact fashion [14].

Although albumin uptake is well established, the intracellular albumin handling and trafficking process remains uncertain. The aim of this study was to define the localization and partners of interaction for proper intracellular albumin handling by applying a Venus-based bimolecular fluorescence complementation, super resolution microscopy, and co-immunoprecipitation.

## 2. Materials and Methods

### 2.1. Cell Culture, Transient Transfection and Crispr/ Cas9-Mediated Knockout of Megalin

Proximal tubule cells (Opossum kidney cells; OK cells) were cultured in 1:1 Dulbecco’s modified Eagles medium/Ham’s F-12 (PAN-Biotech, Aidenbach, Germany), supplemented with 2 mM L-Glutamine (Sigma Aldrich, Taufkirchen, Germany), 5% FCS (PAN-Biotech, Aidenbach, Germany), 5 mM HEPES, 3.0 g/L Na_2_CO_3_ and 100 U/mL penicillin, 100 µg/mL streptomycin (Sigma Aldrich, Taufkirchen, Germany) at 37 °C in a 5% CO_2_ and 95% air atmosphere. BN16 cells (Brown Norway rat yolk sac epithelial cells) were cultured in Dulbecco’s modified Eagles medium, supplemented with 2 mM L-Glutamine (Sigma Aldrich, Taufkirchen, Germany), 10% FCS (PAN-Biotech, Aidenbach, Germany), 100 U/mL penicillin, and 100 µg/mL streptomycin (Sigma Aldrich, Taufkirchen, Germany) at 37 °C in a 5% CO_2_ and 95% air atmosphere. The BN16 cells were a generous gift from Prof. T. Willnow, MDC Berlin.

OK cells were transiently transfected with various plasmids at 0.1 µg/ cm^2^ (see section below) using either a X-fect transfection reagent (ST0159, Takara, Saint-Germain-en-Laye, France) or jetOPTIMUS^®^ (117-01, Polyplus, Illkirch, France), according to the manufacturer’s instructions. For cell differentiation, culture dishes were exposed to gentle shaking by an orbital shaker (1 Hz) for 15 min twice a day. Transfected cells were either fixed at different time points (8, 24, 48 and 72 h) after transfection in 4% PFA /PBS for 10 min, or starved and utilized for an endocytosis assay 72 h after transfection, or collected for co-immunoprecipitation experiments.

Plasmids. Vectors for the Venus-based bimolecular fluorescence complementation, pBiFC-VC155 and pBiFC-VN155, were purchased from Addgene (no. 22011 and 27097). The coding sequence of the rat FcRn (size: 1101 bp) was amplified by PCR and inserted into a pBiFC-VC155 vector between the Sall restriction site (902 bp) and the Kpnl (925) restriction site; the resulting vector was termed FcRn-VC. The coding sequence of the rat megalin mini-receptor 4 (MMR4; ligand-binding domain 4, transmembrane domain and c-terminus; size: 3445 bp, [14]) was inserted into the pBiFC-VN155 vector between the ApaI restriction site (4285 bp) and the Bglll restriction site (74 bp); the resulting vector was termed MMR4-VN. The AMN-VN (amnionless coupled to N-terminal part of Venus) was generated by Vectorbuilder (AMN inserted from 641 bp to 2509 bp; Neu-Isenburg, Germany). Plasmids containing the complete coding sequence of the rat beta2-microgloblin (b2m) and EGFP-tagged rat FcRn were kindly provided by Pamela Björkman; the myc-tagged rat MMR2, myc-tagged rat MMR4, myc-tagged rat LDL2 (Ligand-binding domain 2 and transmembrane (TM) domain of megalin) and myc-tagged rat c-tail (TM domain and cytoplasmic domain of megalin) were used as described in Pohl et al. [14]).

### 2.2. CRISPR/Cas9-Mediated Knockout of Megalin

For the generation of megalin knockout in rat BN16 cells, the CRISPR/Cas9 genome editing technology was applied using the Gene knockout kit v2 (Synthego, Redwood City, CA, USA). Three different sgRNAs targeting exon 2 of the rat megalin gene were designed by Synthego and diluted at a concentration of 60 pmol/µL. BN16 cells were transfected with sgRNAs in the presence of CAS9 nuclease by electroporation using the Neon Transfection System (Thermo Fisher Scientific, Dreieich, Germany), following the recommendations of the manufacturer. After 48 h, cells were minimally diluted and single cell clones were further grown for subsequent mutation analysis. The RNA of respective single cell clones were isolated and a further PCR was amplified and sequenced. The loss of protein was confirmed by a western blot analysis of megalin on megalin-deficient clones. Once the successful deletion of megalin was confirmed, the established method was used to perform a side-by-side analysis of wildtype and megalin-deficient cells. Therefore, transfected cells were plated on a glass coverslip for 72 h and an endocytosis assay was performed, followed by immunohistochemical staining as described below.

### 2.3. Endocytosis Assay

Cells were placed on ice and washed with cold PBS, followed by an incubation with 0.25 mg/mL albumin (A2153, Sigma Aldrich, Taufkirchen, Germany) or with 0.25 mg/mL ß-lactoglobulin (L3908, Sigma Aldrich, Taufkirchen, Germany) at 37 °C for 5 min or 15 min, respectively. Afterwards, cells were placed on ice, washed five times with cold PBS, and fixed in 4% PFA/PBS for 10 min.

### 2.4. Immunocytochemistry

For immunocytological stainings, cells plated on matrigel matrix- (354234, Corning, Kaiserslautern, Germany) coated glass coverslips were used. Fixed cells were permeabilized with 0.5% Triton X-100, blocked with 5% dry milk/PBS for 30 min and stained with primary the antibody overnight at 4 °C. After washing, the cells were incubated with suitable secondary antibodies for 2 h at room temperature. The nuclei were stained using 4′,6-diamidino-2-phenylindole (DAPI, Thermo Fisher Scientific, Dreieich, Germany) and cells were mounted with Abberior Mount Liquid Antifade (MM-2009-2X15ML, Abberior GmbH, Göttingen, Germany).

Antibodies. Rabbit anti-GM130 (ab52649, Abcam, Cambridge, UK), mouse anti-EEA1 (610457, BD Biosciences, Heidelberg, Germany), mouse anti-clathrin (CBL188, Millipore, Darmstadt, Germany), rabbit anti-Rab11 (71-5300, Invitrogen, Darmstadt, Germany), goat anti-lamp1 (sc-8098, Santa Cruz Biotechnology, Dallas, TX, USA), guinea pig anti-megalin [15], goat anti-calnexin (NBP1-3774, Novus, Bio-Techne, Wiesbaden, Germany), mouse anti-FcRn (sc-393064, Santa Cruz Biotechnology, Dallas, TX, USA) and rabbit anti-FcRn (sc-66893, Santa Cruz Biotechnology, Dallas, TX, USA) were used. Secondary antibodies used were coupled with StarRED, Star580 or Alexa488; all of them were suitable for STED microscopy. They were either purchased from Abberior GmbH (anti-rabbit Star580 (ST580-1002), anti-mouse Star580 (ST580-1001), anti-guinea pig StarRed (STRED-1006), anti-mouse StarRed (STRED-1001)), or donkey-derived secondary antibodies (Dianova) were coupled with Star580 NHS ester (ST580-0002, Abberior GmbH, Göttingen, Germany) or StarRed NHS carbonate (STRED-0002) in our lab according to the manufacturer’s instructions. Rhodamine-coupled phalloidin (R415, Invitrogen) was used to show cell borders.

### 2.5. Super-Resolution Microscopy and Confocal Microscopy

Images were acquired using Facility Line (Abberior Instruments, Göttingen, Germany) with Olympus IX83 microscope (Hamburg, Germany) and Imspector software (Abberior Instruments, Göttingen, Germany). Images were de-convoluted using Huygens Professional Software (version 20.10, Scientific Volume Imaging B.V., Hilversum, The Netherlands). CMLE algorithm was performed using a quality change threshold of 0.1 and a maximum of 40 cycles. Co-localization was analyzed using a colocalization analyzer of Huygens software. The mean values of the Pearson correlation coefficient (PCC) were evaluated statistically.

### 2.6. Co-Immunoprecipitation (Co-IP) and Western Blot

Transiently transfected cells or mouse kidneys were lysed/sonicated in Co-IP buffer [20 mM Tris-HCl, 2 mM EDTA, 1% NP40 supplemented with proteinase inhibitor cocktail (cOmplete, EDTA-free, Roche Diagnostics, Basel, Schweiz) and phosphatase inhibitors (10 mM sodium phosphate, 100 mM sodium fluoride, 1 mM sodium orthovanadate)]. Cell lysates or kidney homogenates were centrifuged at 21,500× *g* for 30 min at 4 °C. For the samples to be tested at acidic conditions, the pH of their lysates was adjusted to 5.5. For blocking the ligand-binding site of LDL2, lysates were incubated with 10 µg (320 nM) receptor associated protein RAP for 30–60 min at 4 °C. Afterwards, cells lysates were incubated with mouse anti-Myc antibody (SAB4700447, Sigma Aldrich, Taufkirchen, Germany) or mouse anti-Flag antibody (negative control; 66008-3-Ig, Proteintech, Planegg-Martinsried, Germany), and kidney homogenates were incubated with a guinea pig anti-megalin antibody [16], and placed on an end-over-end rotator for 2 h at 4 °C. The lysates/antibody mixtures were further incubated with magnetic beads (Pierce protein A/G magnetic beads, Thermo Fisher Scientific, Dreieich, Germany) for an additional 2 h at 4 °C. Magnetic beads were washed five times with Co-IP buffer, and incubated with Laemmli elution buffer for 5 min at 95 °C. Eluates were analyzed via SDS-PAGE, performed on 10–15% polyacrylamide gels and with subsequent immunoblotting onto nitrocellulose membranes. Membranes were incubated either with rabbit anti-Myc (16286-1-AP, Proteintech, Planegg-Martinsried, Germany), goat anti-FcRn (AF6775, R&D systems, Bio-Techne, Wiesbaden, Germany), or with rabbit anti-megalin [17] antibody overnight at 4 °C. Membranes were incubated with suitable HRP-labeled secondary antibodies for 2 h at room temperature. Immunoreactive bands were detected by chemiluminescence using Immobilon Western HRP substrate (Millipore, Darmstadt, Germany) in combination with the chemiluminescence imaging system Fusion SL (Peqlab, Erlangen, Germany).

### 2.7. Statistical Data Analysis

Statistical comparisons were performed with the GraphPad Prism Software Package 5 (GraphPad Software, La Jolla, CA, USA) using the Kruskal-Wallis test, the Mann-Whitney U test, or Student’s *t*-test. *p* values of <0.05 were judged statistically significant. Asterisks are used in the figures to explicitly demonstrate the statistical significance (* *p* < 0.05; ** *p* < 0.01; *** *p* < 0.001).

## 3. Results

### 3.1. FcRn Interacts with Megalin in the Endosomal Compartment and Increases after Induction of Endocytosis

**Figure 1:**
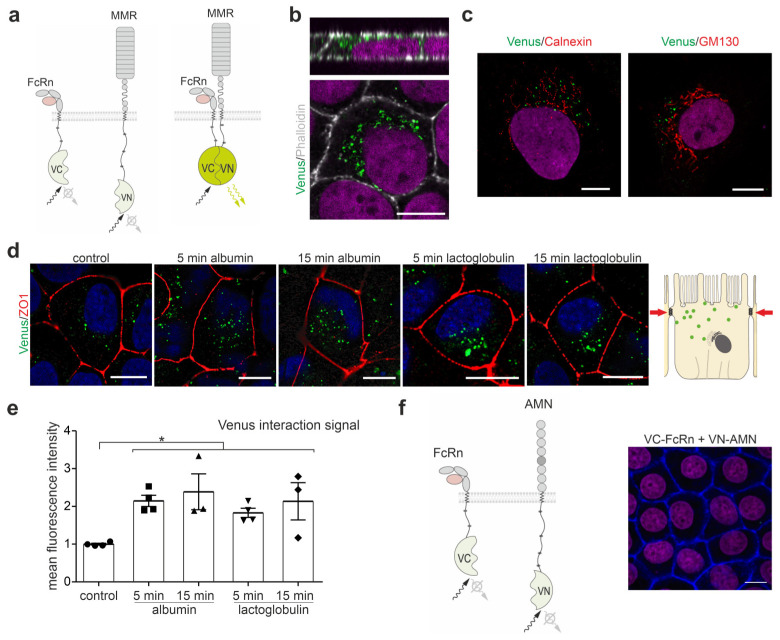
Megalin interacts with FcRn. (**a**) Cartoon of the Venus-based bimolecular fluorescence complementation system used. C-terminal part of the Venus protein is fused to FcRn (VC-FcRn), and the N-terminal part of the Venus protein to the megalin mini receptor MMR4 (VN-MMR4). Fluorescence is generated only when both proteins, FcRn and MMR4, are in close vicinity to each other. (**b**) Labeling of VC-FcRn and VN-MMR4 co-transfected opossum kidney cells (OKC) for 72 h with phalloidin to stain actin fibers, i.e., to visualize the brush border. Nuclei are counterstained with DAPI. Magnification scale bar = 10 µm. (**c**) Labeling of VC-FcRn and VN-MMR4 co-transfected opossum kidney cells (OKC) for 8 h with calnexin (ER marker) or GM130 (Golgi marker). Magnification scale bar = 10 µm. (**d**) Venus fluorescence of OKC co-transfected with VC-FcRn and VN-MMR4 for 72 h and endocytosis induction using albumin or lactoglobulin for 5 and 15 min. Cells were stained with anti-ZO-1 to mark tight junctions and DAPI to mark nuclei. Magnification scale bar = 10 µm. (**e**) Densitometric evaluation of Venus fluorescence intensity per cell area. >150 cells of n = 3–4 independent experiments, Kruskal-Wallis test, * *p* < 0.05 was considered significant. (**f**) (*left*) Cartoon of the Venus-based bimolecular fluorescence complementation system used. The C-terminal part of the Venus protein is fused to FcRn (VC-FcRn) and N-terminal part of the Venus protein to amnionless (AMN, VN-AMN). (*right*) OKC double transfected with VC-FcRn and VN-AMN. Cells were counterstained with phalloidin to stain actin fibers and DAPI to stain nuclei. No Venus fluorescence was detected. Magnification scale bar = 10 µm.

To identify whether megalin and FcRn interact, and to localize the interaction to a specific location within the living cell, we used the bimolecular fluorescence complementation method; this uses split Venus fragments to detect the protein-protein interaction, as illustrated in Figure 1a. In the 72 h after the transient transfection of both VC-coupled FcRn and VN-coupled megalin mini receptor 4 (MMR4), an interaction was encountered in endosomal vesicles underneath the brush border membrane (Figure 1b). Occasionally, an interaction was observed in microvilli bundles of cells co-stained with phalloidin for actin bundles of brush border microvilli. To address the question of whether the interaction of FcRn and megalin occurs early upon protein production or assembly in the Golgi apparatus, the co-transfection of both the VN-coupled MMR4 and VC-coupled FcRn was analyzed 8 h after transient transfection. Venus fluorescence was identified in vesicles outside the endoplasmic reticulum (ER), as co-stained with calnexin marking the ER, and outside the Golgi apparatus, as co-stained with GM130 an established marker for the Golgi apparatus (Figure 1c); this indicated that the interaction between FcRn and megalin occurs solely in the endosomal compartment beyond the Golgi apparatus. To analyze whether the induction of endocytosis impacts the colocalization of megalin and FcRn, we applied albumin, a ligand for both receptors or lactoglobulin, a ligand only for megalin; these were applied for 5 min and 15 min, respectively, and we determined the amount of Venus fluorescence per cell 72 h after the transient transfection of VN-coupled MMR4 and VC-coupled FcRn. Albumin and, surprisingly, lactoglobulin-induced endocytosis strongly increased the colocalization between the megalin mini receptor and FcRn after 5 and 15 min (Figure 1d,e).

To analyze whether AMN might interact with FcRn also, VN-coupled AMN and VC-coupled FcRn were co-transfected. No specific Venus fluorescence signal was observed at 24, 48 and 72 h after transient transfection (Figure 1f).

### 3.2. Megalin and FcRn Interact within Their Extracellular Domains

**Figure 2:**
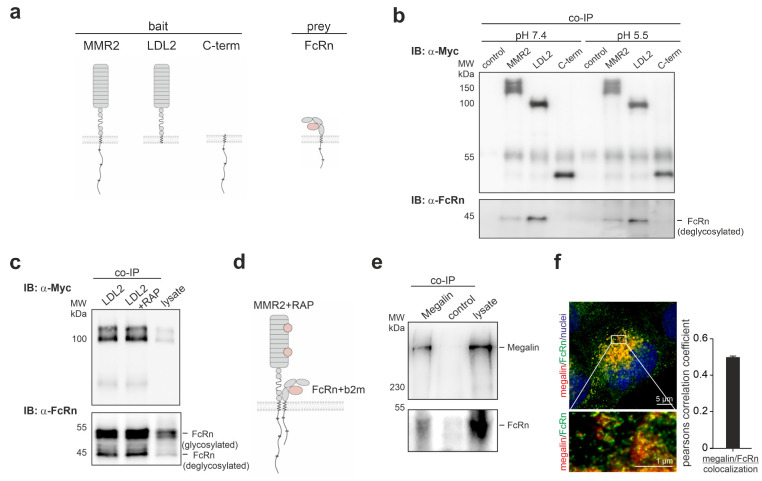
FcRn binds to the extracellular domain of megalin outside of the ligand-binding domain. (**a**) Cartoon of truncated megalin domains (MMR2-megalin mini receptor 2; LDL2-extracellular domain of MMR2 without C-terminal domain; C-term-C-terminal domain of megalin) and FcRn used for co-immunoprecipitation. (**b**) Co-immunoprecipitation (Co-IP) of truncated megalin domains and FcRn, using lysates of cells transiently transfected with either myc-tagged MMR2, myc-tagged LDL2, and myc-tagged c-terminus and FcRn at pH = 7.4 and pH = 5.5. FcRn co-immunoprecipitants with MMR2 and LDL2. Interaction is similar at neutral and acidic pH conditions. (**c**) Co-immunoprecipitation of the extracellular domain of megalin mini receptor (LDL2) and FcRn in combination with receptor associated protein (RAP), using lysates of cells transiently transfected with myc-tagged LDL2 and FcRn, and 10 µg RAP. Addition of RAP did not inhibit binding of FcRn to megalin. (**d**) Summarizing cartoon demonstrating the interaction of the extracellular domains of megalin and FcRn lying outside the ligand binding domains of megalin. (**e**) Co-immunoprecipitation of megalin and western blot of megalin and FcRn of mouse kidney homogenate. Endogenous FcRn binds to megalin in vivo. (**f**) STED image and Pearson correlation coefficient of endogenous megalin and FcRn in rat BN16 cells showing high colocalization of both proteins.

To verify the binding of megalin and FcRn biochemically, and to identify the protein domain for interaction, co-immunoprecipitation was performed using either the megalin mini receptor MMR2, the extracellular domain of MMR2 (LDL2), or the c-terminus of megalin together with FcRn, as illustrated in Figure 2a. FcRn was found to bind to MMR2 and LDL2 and not to the c-terminus of megalin, pointing to a binding of both extracellular domains (Figure 2b, Supplemental Figure S2). Because it was suggested that both proteins may interact in endosomal vesicles with lower pH, we further investigated the binding properties at a pH of 5.5. pH 5.5 neither affected the binding between FcRn and MMR2, nor between FcRn and LDL2. Interestingly, the affinity of FcRn to MMR2 was always lower than to LDL2, suggesting that the c-terminal domain of megalin negatively influences the interaction between both proteins. Based on the extracellular interaction between FcRn and MMR2, we analyzed the possibility of binding to the ligand binding domain. The receptor associated protein (RAP) is a protein known to bind and block the ligand binding domain of megalin intracellularly, and to prevent unspecific ligand binding. The addition of 10 µg RAP did not alter binding between both extracellular domains of FcRn and MMR2, suggesting that binding of FcRn to the extracellular domain of megalin is located outside the ligand binding domains (Figure 2c). The results of the interaction between megalin and FcRn are illustrated in Figure 2d. To analyze the colocalization and interaction of megalin and FcRn endogenously, we performed a co-immunoprecipitation and STED imaging of endogenous megalin and FcRn. Co-immunoprecipitation, using mouse kidney lysate, demonstrated that both megalin and FcRn interact and bind to each other (Figure 2e). Furthermore, we performed STED imaging of megalin and FcRn in BN16 cells, revealing high values for the Pearson correlation coefficient for both proteins, supporting a colocalization in vivo (Figure 2f).

### 3.3. Megalin and FcRn Interact in Clathrin Vesicles upon Endocytosis Induction

**Figure 3:**
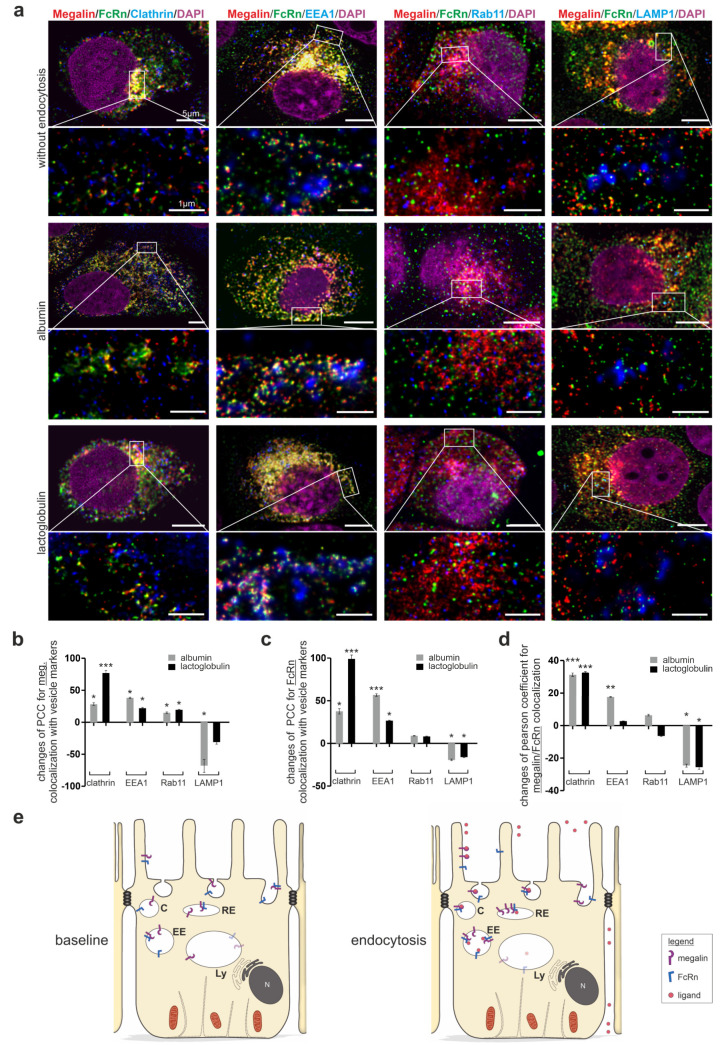
Endocytosis induced movement of megalin and FcRn into clathrin vesicles and early endosomes bypassing lysosomes. (**a**) Quadruple staining of FcRn, megalin, with either clathrin for clathrin vesicles, with EEA1 for early endosomes, with Rab11 for recycling endosomes and with LAMP1 for late endosomes/lysosomes without endocytosis (starved cells) or induction of endocytosis by albumin as ligand for megalin and FcRn or by lactoglobulin, a ligand only for megalin. Nuclei are stained with DAPI. Higher resolution STED images of inset are shown below for each respective image. Magnification scale bar = 5 µm and 1 µm as indicated. Relative changes in colocalization of megalin with respective vesicle marker (**b**), FcRn with respective vesicle marker (**c**) or megalin with FcRn (**d**) upon endocytosis induction by albumin or lactoglobulin using the Pearson correlation coefficient. Here, >120 cells of n = 4–5 independent experiments, Mann-Whitney U test, * *p* < 0.05, ** *p* < 0.01 and *** *p* < 0.001 was considered significant. (**e**) Summarizing cartoon showing the expression and localization of megalin and FcRn at baseline (starved cells), after induction of endocytosis with higher expression, colocalization of megalin and FcRn in clathrin vesicles, early endosomes, reduced expression, and colocalization in lysosomes. C, clathrin vesicle; EE, early endosome; RE, recycling endosome; Ly, lysosome.

Because FcRn is known to bind its ligands at a pH lower than 7.4, we aimed to identify in which vesicles FcRn binds to megalin for ligand transfer. To fulfil this aim, super resolution microscopy of FcRn, megalin and the respective endocytic vesicle marker, clathrin for clathrin vesicles, EEA1 for early endosomes, Rab11 for recycling endosomes, and LAMP1 for late endosomes/lysosomes, was performed upon the induction of the endocytosis; this used albumin, as it is the known ligand for megalin and FcRn, and lactoglobulin, as it is the known ligand for megalin (Figure 3a). At the baseline, megalin and FcRn show the highest colocalization within Rab11-positive vesicles, and, therefore, residence in recycling endosomes, as published previously; data not shown.

For megalin, the induction of endocytosis by albumin or lactoglobulin induced higher values of the Pearson colocalization coefficient for megalin with clathrin, EEA1 and Rab11 (Figure 3a,b). Reduced values of the Pearson correlation coefficient were identified for megalin with LAMP1 upon albumin incubation. These results suggest that albumin and lactoglobulin induce a movement of megalin into clathrin vesicles, early endosomes, and recycling endosomes; in addition, for albumin, there is a lower movement into lysosomes.

For FcRn, the induction of endocytosis by albumin or lactoglobulin induced higher values of the Pearson correlation coefficient with clathrin and EEA1 (Figure 3a,c). Reduced values of the Pearson correlation coefficient were identified for FcRn with LAMP1 upon albumin or lactoglobulin incubation, suggesting the movement of FcRn into clathrin vesicles and early endosomes, which is lower into lysosomes.

Additionally, the values of the Pearson correlation coefficient of megalin and FcRn upon endocytosis induction with albumin and lactoglobulin was analyzed (Figure 3d). Albumin and lactoglobulin induced a significantly higher colocalization of megalin, FcRn and either clathrin or EEA1; they also reduced colocalization with LAMP1, suggesting that both ligands induce a colocalization in clathrin vesicles and early endosomes, but a lower movement into lysosomes. For better visualization, the results are illustrated in Figure 3e.

In addition, ovalbumin was presented earlier as a protein, which does not bind to megalin in OKC [18]. For verification of our experiments, we performed additional experiments using ovalbumin as a ligand for endocytosis induction. In comparison to albumin or lactoglobulin, the Pearson correlation coefficient of megalin and FcRn in clathrin vesicles was even reduced (Supplemental Figure S1). These results support the specificity of albumin and lactoglobulin induced endocytosis in colocalizing megalin and FcRn in clathrin vesicles.

### 3.4. Megalin Knockout Impairs FcRn Trafficking and Function

**Figure 4:**
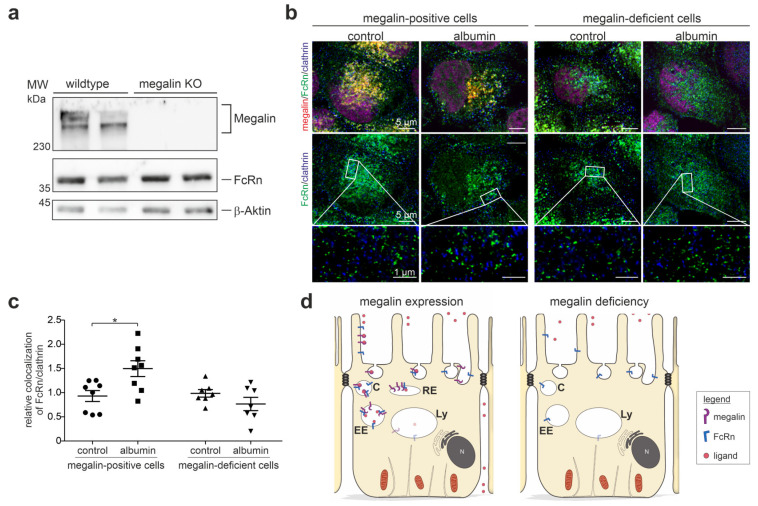
Megalin deficiency prevents FcRn trafficking. (**a**) Western blot of megalin and FcRn on wildtype and megalin-deficient cells by CRISPR/Cas9. Successful deletion of megalin is confirmed, the abundance of FcRn remains unaltered. Beta-actin is used as house keeping control. (**b**) Quadruple staining of FcRn, megalin, and clathrin for clathrin vesicles, at baseline (control) or induction of endocytosis by albumin. Nuclei are stained with DAPI. Higher resolution STED images of inset are shown below for each respective image. Magnification scale bar = 5 µm and 1 µm as indicated. (**c**) Relative changes in colocalization of FcRn and clathrin upon endocytosis induction by albumin using Pearson correlation coefficient. In this study, >150 cells of n = 7–8 independent experiments, student’s *t*-test, * *p* < 0.05 was considered significant. (**d**) Summarizing cartoon showing wildtype (megalin-positive) cells upon albumin-induced endocytosis movement of FcRn into clathrin vesicles, which is prevented in megalin-deficient cells. C, clathrin vesicle; EE, early endosome; RE, recycling endosome; Ly, lysosome.

To analyze the impact of megalin on FcRn trafficking, rat yolk sac epithelial BN16 cells, which nicely express both proteins [19,20], were used for deletion of megalin by the CRISPR/Cas9 method. Megalin was successfully deleted, as shown by the western blot analysis of megalin expression (Figure 4a, Supplemental Figure S3). The baseline abundance of FcRn remained unaltered upon megalin deletion. To address the question of whether megalin is needed for proper trafficking of FcRn into clathrin vesicles upon albumin-induced endocytosis, we analyzed wildtype and megalin-deficient cells on the same dish and induced endocytosis of albumin for 5 min (Figure 4b). STED-imaging and quantification of the Pearson correlation coefficient revealed that wildtype cells respond to albumin-induced endocytosis with a significantly higher colocalization coefficient between FcRn and clathrin, compared to the control (starved) cells (Figure 4b,c). In contrast, in megalin-deficient cells, the Pearson correlation coefficient of FcRn and clathrin remained unchanged upon endocytosis induction by albumin. These results suggest that megalin binds FcRn for trafficking into clathrin vesicles upon endocytosis and is, therefore, a prerequisite to exert proper FcRn function, as illustrated in Figure 4d.

## 4. Discussion

In summary, we found that megalin and FcRn specifically interact within their extracellular domain outside the ligand-binding domain of megalin. This interaction occurred at a neutral and acidic pH to similar extents. The interaction of both proteins was encountered in vesicles of the endosomal compartment, and, to lesser extent, at the plasma membrane. This interaction can be induced upon megalin initiated receptor-mediated endocytosis. We can further show that megalin, as master regulator of proximal tubule function, also controls FcRn trafficking into the clathrin vesicle for endocytic uptake and ligand binding as a prerequisite for the transcytosis of albumin.

The Venus-based bimolecular fluorescence complementation system, used to tag the megalin mini receptor or FcRn, has a center-to-center fluorophore proximity in crystals of 2.5 nm [21], enabling the localization of interaction within a living cell. However, a disadvantage is that, once the Venus protein is formed, it remains together and is further distributed within the cell, depending on the destination of the proteins used for the Venus-tag [22]. Therefore, the use of STED microscopy, allowing a similar resolution of approximately 15 nm, is necessary to add valuable information regarding the localization of proteins within a cellular compartment.

FcRn is a MHC class-I-related receptor consisting of a heterodimer with a heavy (α)-chain and β2-microglubulin light chain, common to all MHC class I molecules. It is widely distributed throughout the body and was shown to bind IgG in a 2:1 ratio and albumin in a stoichiometry of 1:1 at separate binding sites [23]. The binding is known to occur only at a mild acidic pH of 5.0 to 6.5. The cytoplasmic tail of FcRn was demonstrated to contain several serine phosphorylation sites, with the phosphorylation of S-313 being important for apical to basal transcytosis, allowing FcRn to be diverted from the apical recycling pathway into the basolateral directed transcytosis pathway [24]. Interestingly, basolateral to apical transcytosis seemed to be independent of the existence of the FcRn cytoplasmic tail. The cytoplasmic tail of FcRn further contains a dileucine and a tryptophan endocytosis motif for adaptor protein-2 (AP-2) binding and endocytosis [25], and a calcium-dependent calmodulin binding site for regulating transcytosis and FcRn half-life [26]. The signal, however, which orchestrates FcRn trafficking, e.g., from the apical membrane, to the endosomal compartment, and to the basolateral membrane, remains unknown. We found that megalin interacts with FcRn at its extracellular domains, and, upon endocytosis induction, the protein interaction between megalin and FcRn augmented and occurred early in the endocytic pathway in clathrin vesicles. At the same time, this interaction induced a lower movement of megalin and FcRn into lysosomes, suggesting that the induction of endocytosis induced a higher demand of the receptors reducing their degradation. We further detected that megalin-deficiency impaired the trafficking of FcRn into clathrin vesicles, suggesting that megalin may be responsible for orchestrating the FcRn endocytosis, and, therefore, early events in FcRn-mediated transcytosis.

Megalin is one of the main receptors for clathrin-mediated endocytosis in the proximal tubule. It is a large transmembrane protein belonging to the family of low-density lipoprotein receptors. The megalin molecule contains a large N-terminal extracellular domain containing four areas rich in cysteine for ligand binding, several epidermal growth factor repeats, and cysteine-poor fragments containing YWTD motifs that separate the ligand binding regions [27]. We identified the location of the interaction between MMR4 and FcRn between both extracellular domains outside the ligand-binding domain since the addition of RAP did not alter their interaction. Therefore, we assume an interaction between FcRn and the YWTD spacer region, and EGF-like repeats of megalin. In comparison to the cytoplasmic tail of FcRn, the cytoplasmic tail of megalin harbors three NPXY motifs for endocytosis. It further contains several binding sites for adaptor proteins, including DAB2, for clathrin-mediated endocytosis, autosomal recessive hypercholesteremia (ARH), for reserving megalin in recycling endosomes, oculocerebrorenal syndrome protein 1, an inositol 5-phosphatase required for endocytosis, and the small guanosine triphosphatase (GTPase) Rab11, resulting in the diminished apical delivery of megalin [28]. In addition, the cytoplasmatic tail of megalin is highly phosphorylated by glycogen synthase kinase 3 (GSK3β), slowing down the recycling from endosomes to the plasma membrane [28]. In our experiments, we observed the negative influence of the cytoplasmic domain of megalin on the affinity of FcRn to megalin. Signaling events phosphorylating the cytoplasmic domain may alter the extracellular binding affinity of megalin to FcRn. In comparison, the cytoplasmic domain of FcRn is relatively small, supposing that the interaction with megalin is necessary for intracellular trafficking upon endocytosis; however, this contains an AP-2 motif for the initial event of endocytosis. Megalin may guide FcRn from the apical plasma membrane into the early endosomes for its degradation, but this is lower for lysosomes. We have also observed a similar increase in the Pearson correlation coefficient of both proteins, megalin and FcRn, with endocytic compartment proteins during endocytosis. We therefore propose that megalin orchestrates FcRn endocytosis and intracellular trafficking as early events of cellular transcytosis.

Cubilin is another receptor for albumin highly expressed in the renal proximal tubule and elsewhere in the body. Cubilin does not have a transmembrane domain and, therefore, must interact with AMN or megalin for internalization [27]. We also investigated a possible interaction between FcRn and AMN using the split Venus system, however no interaction was observed between them at various time points after transient transfection. Therefore, CUBAM mediated albumin uptake may exert a different cellular function than cubilin-megalin-FcRn-mediated albumin uptake. The cytoplasmic domain of AMN has a FXNPXF motif mediating the sequestration of CUBAM by interacting with DAB2 and ARH [27]. However, in comparison to the C-terminal domain of megalin, the cytoplasmic domain of AMN contains far fewer motifs for signaling and trafficking, suggesting that megalin is the main scavenger receptor orchestrating FcRn endocytosis; it is, therefore, also the early event in cellular transcytosis process.

The signaling process and proteins involved in enrolling endocytosed proteins for either transcytosis or lysosomal degradation need to be further explored.

## Data Availability

The data is contained within the article.

## Supplementary Materials

The following supporting information can be downloaded at: https://www.mdpi.com/article/10.3390/cells12010053/s1, Figure S1: Ovalbumin does not induce a colocalization of megalin and FcRn in clathrin vesicles; Figure S2: Original blots to Figure 2 and supportive data; Figure S3: Original blots to Figure 4.
