# Supplementary material for: Megalin Orchestrates FcRn Endocytosis and Trafficking
Source: Cells. 2022 Dec 22;12(1):53. doi: 10.3390/cells12010053 (PMC9818419; doi:10.3390/cells12010053)
Supplement: Supplementary file 1 [file cells-12-00053-s001.zip › cells-2019895-Supplemnetary Materials.pdf]

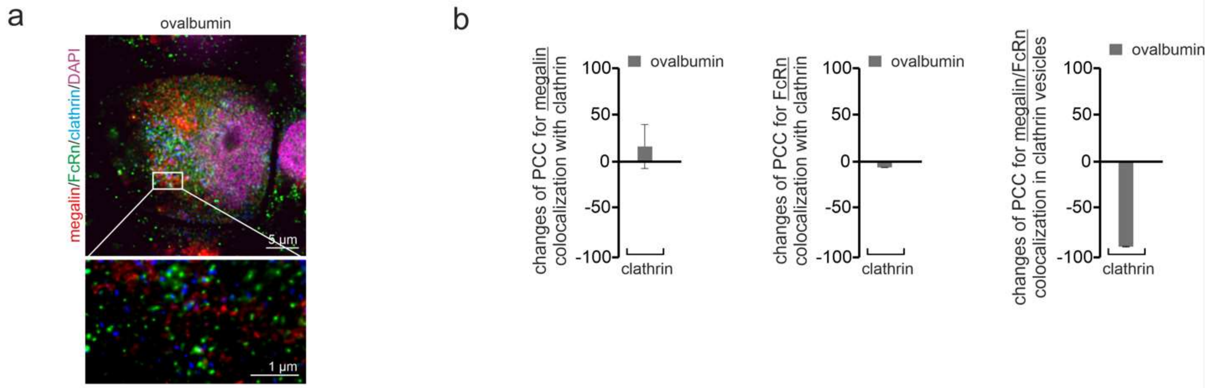

Figure S1. Ovalbumin does not induce a colocalisation of megalin and FcRn in clathrin vesicles. (**a**) Quadriple staining of FcRn, megalin, clathrin for clathrin vesicles, and DAPI for nuclei of OKC after incubation with 0.25 mg/ml ovalbumin for 5 min. Higher resolution STED images of inset are shown below. Magnification scale bar = 5 um and 1 um as indicated. (**b**) Relative changes in colocalization of megalin with clathrin, FcRn with clathrin and megalin and FcRn in clathrin vesicles using Pearson correlation coefficient. >80 cells of n = 2 independent experiments.

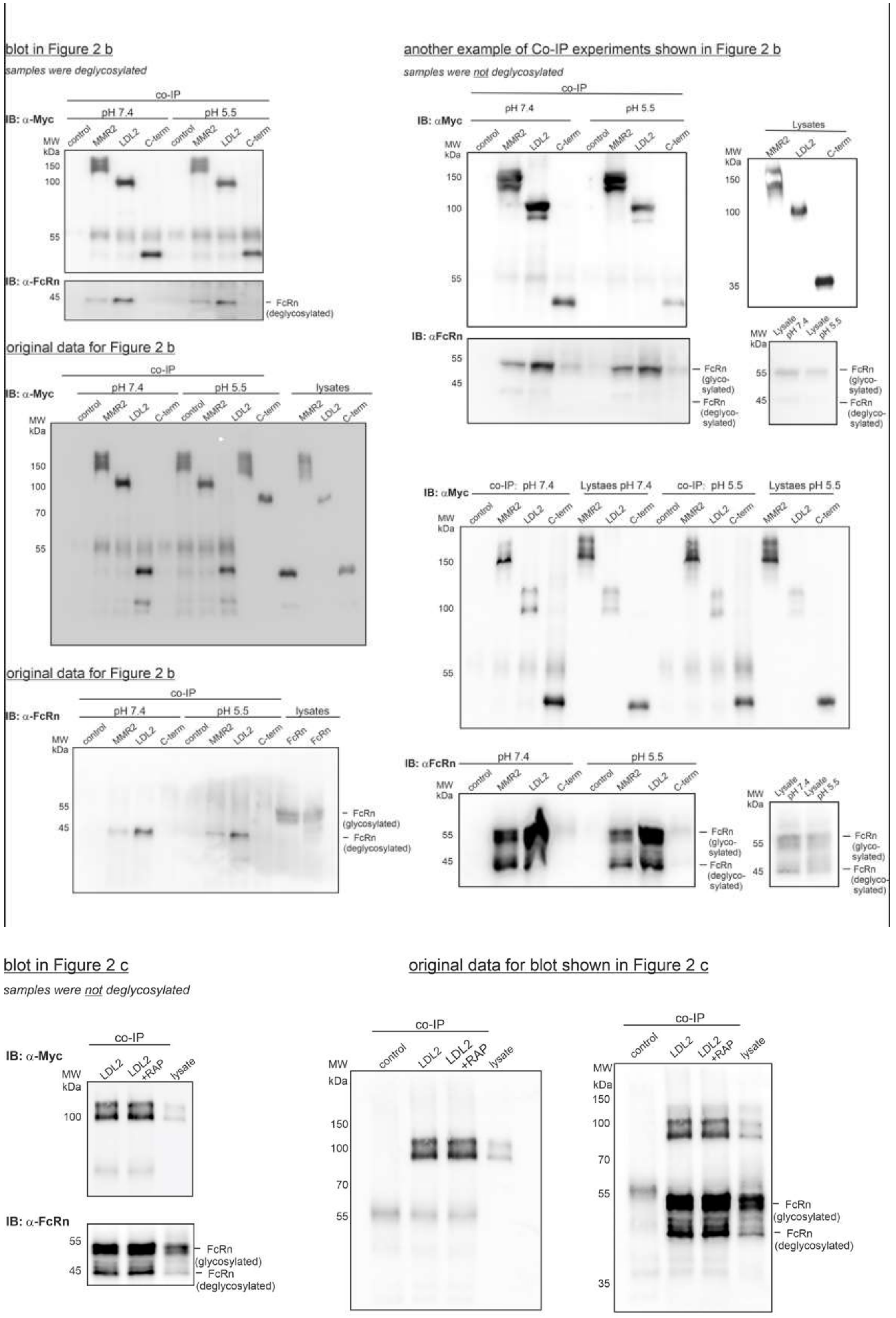

Figure S2. Original blots to Figure 2 and supportive data.

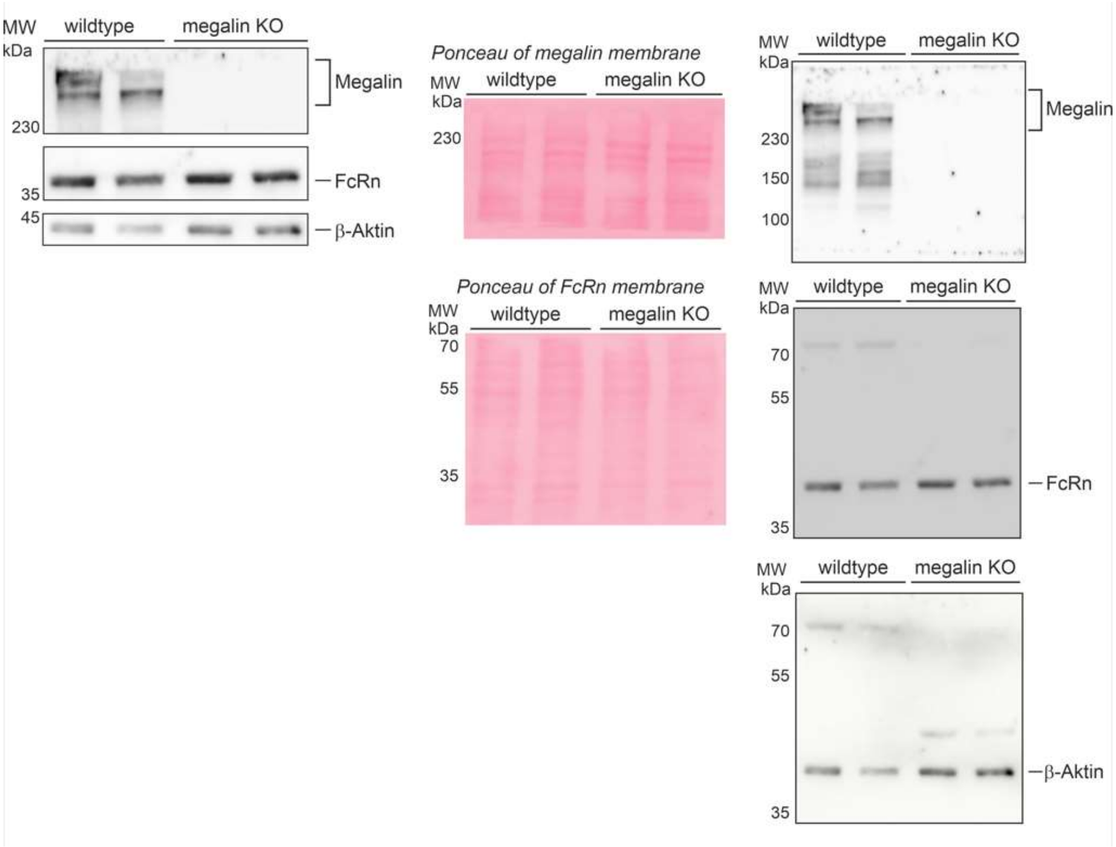

Figure S3. Original blots to Figure 4.
